# Association of Whole Blood Fatty Acids and Growth in Southern Ghanaian Children 2–6 Years of Age

**DOI:** 10.3390/nu10080954

**Published:** 2018-07-24

**Authors:** Mary Adjepong, William Yakah, William S. Harris, Esi Colecraft, Grace S. Marquis, Jenifer I. Fenton

**Affiliations:** 1 Department of Food Science and Human Nutrition, Michigan State University, 469 Wilson Rd, East Lansing, MI 48824, USA; adjepong@anr.msu.edu (M.A.); yakahwil@msu.edu (W.Y.); 2 Sanford School of Medicine, University of South Dakota and Omega Quant Analytics, LLC, Sioux Falls, SD 57106, USA; bill@omegaquant.com; 3 Department of Nutrition and Food Science, University of Ghana, P.O. Box LG 25, Accra 00233, Ghana; colecraft_s@hotmail.com; 4 School of Human Nutrition, McGill University, 21,111 Lakeshore Rd, Sainte Anne de Bellevue, QC H9X 3V9, Canada; grace.marquis@mcgill.ca

**Keywords:** omega-3 index, Ghana, long chain polyunsaturated fatty acids (LCPUFA), stunting, undernutrition, fish

## Abstract

In Ghana, stunting rates in children below 5 years of age vary regionally. Dietary fatty acids (FAs) are crucial for linear growth. The objective of this study was to determine the association between blood FAs and growth parameters in southern Ghanaian children 2–6 years of age. A drop of blood was collected on an antioxidant treated card and analyzed for FA composition. Weight and height were measured and z-scores calculated. Relationships between FAs and growth were analyzed by linear regressions and factor analysis. Of the 209 subjects, 22% were stunted and 10.6% were essential FA deficient (triene/tetraene ratio > 0.02). Essential FA did not differ between stunted and non-stunted children and was not associated with height-for-age z-score or weight-for-age z-score. Similarly, no relationships between other blood fatty acids and growth parameters were observed in this population. However, when blood fatty acid levels in these children were compared to previously reported values from northern Ghana, the analysis showed that blood omega-3 FA levels were significantly higher and omega-6 FA levels lower in the southern Ghanaian children (*p* < 0.001). Fish and seafood consumption in this southern cohort was high and could account for the lower stunting rates observed in these children compared to other regions.

## 1. Introduction

Growth stunting, a condition of impaired development, is a strong indicator of chronic malnutrition and a major global nutritional challenge in Ghana [1]. The 2014 Ghana Demographic Health Survey (GDHS) reported that 19% of children under five years of age were stunted [1]. Stunting typically becomes permanent once established and may be caused by poor maternal diet, frequent childhood infections and inadequate nutrient intake [2]. There have been numerous interventions implemented to curb stunting including dietary supplementation and fortification of vitamins and minerals. Recent fatty acid (FA) supplementation studies in Ghana demonstrated that lipid-based supplements increased hemoglobin (Hb) levels of pregnant women as well as linear growth in children [3,4]. However, these studies did not characterize the FA content in whole blood. Further, research investigating the relationship between circulating FA levels and growth is scarce in this population. 

FAs have numerous physiological functions in human growth and development. For example, polyunsaturated FA (PUFA)-derived eicosanoids can activate transcriptional factors to influence stem cell proliferation and differentiation [5] and essential FAs (EFAs) help build structural barriers to prevent energy loss by the accumulation of linoleic acid (LA) into the stratum corneum [6]. These FAs can be metabolized into molecules with specificity for receptors whose signal transduction pathway results in changes to linear growth [6]. Essential FAs are those FAs which cannot be produced in the body because humans lack the specific enzymes required for their *de novo* biosynthesis. Linoleic acid (C18:2n6, LA) and alpha-linolenic acid (C18:3n3, ALA) are the two main EFAs in the human diet [7,8]. These omega-6 (n-6) and n-3 FAs are substrates for the desaturases (delta-5 and delta-6 desaturase) and elongases that produce the long chain (LC) PUFAs such as eicosapentaenoic acid (EPA), docosahexaenoic acid (DHA), arachidonic acid (AA), among others [9]. The elongase enzymes usually prefer n-6 and n-3 FAs as substrates but in their absence, they convert n-9 FAs into some LC-PUFA such as Mead acid, the appearance of which is a hallmark of EFA deficiency [10,11]. More specifically, when an individual’s diet is deficient of EFAs, oleic acid (C18:1n9), a non-essential n-9 FA, is converted to Mead acid (C20:3n9) [12]. Mead acid is then incorporated into phospholipids, cholesterol esters, triglycerides and non-esterified free FAs [13,14]. Therefore, in EFA deficiency, there are elevated levels of Mead acid with a decrease in the production of other EFA metabolites such as AA. The ratio of Mead acid (3 double bonds, triene) and AA (4 double bonds, tetraene), termed as triene-to-tetraene ratio (T/T ratio), is a functional biomarker for essential fatty acid deficiency (EFAD [15,16,17]. EFAD is defined by a T/T ratio >0.02 in plasma samples [15,16] and also established when Mead acid [11] levels are above 0.4% in red blood cells (RBCs) [17] and 0.21% in plasma [15]. 

Child undernutrition is prevalent in Ghana due to low dietary diversity and poor infant and young child feeding (IYCF). Only 15% of breastfed Ghanaian children met minimum standards of IYCF practices with respect to both dietary diversity and feeding frequency [1]. Additionally, complementary foods are often low in essential macro- and micronutrients [18]. Evidence of the poor quality diet can be noted, in part, in the high prevalence of anemia in all ten regions of the country with a greater than 40% prevalence of anemia [19], reflecting a severe category of public health significance of anemia as categorized by the World Health Organization (WHO) [20]. These dietary intake patterns coupled with poor infant feeding practices could increase EFAD in infants and young children. The dietary sources of the parent EFAs in Ghanaian diets are peanut (LA-rich), melon seeds (LA-rich) and soy bean (rich in both ALA and LA) [21]. However, insufficient consumption of foods that are good sources of EFAs may be leading to growth impairment. 

Recently, some small-scale studies in Ghana using lipid-based supplementation showed an increase in linear growth in Ghanaian children [3,4], however, blood assessment of FA levels in Ghanaian children have not been extensively characterized and the prevalence of EFAD has been poorly documented. Considering the significance of FAs in growth and development, the objective of this study was to assess blood FA levels in 2–6-year-old Ghanaian children and their association with growth. 

## 2. Materials and Methods

### 2.1. Study Setting

The study was conducted in the Upper Manya Krobo district, the district capital of which is Asesewa, located in the south-eastern region of Ghana. The district covers 859.1 sq. km with a population of 72,092. The district comprises of 13,111 households with an average household size of 4.6 persons per household. The rainfall ranges from 900 mm to 1500 mm with temperature ranging from 26 °C to 32 °C. The district lies within the semi-deciduous forest and savanna zone. Palm, dawadawa, mango, neem and acacia interspersed with shrubs are the major vegetation in the district. Agriculture, forestry and fishing constitute the largest industry in the locality employing over 72% of the workforce aged 15 years and above. Boreholes, tube well, pumps, rivers and streams constitute the sources of water supply of household for domestic purposes. Metal sheet is the main roofing material for housing (87.9%). Illiteracy level is high with 33.3% of all inhabitants 11 years and above having no education. The community has one hospital, 3 maternity homes, 4 health centers and 15 Community Health Posts [21]. 

### 2.2. Subjects for the Present Study

Children between 2 and 6 years of age residing in communities in the Upper Manya Krobo district, Ghana, were recruited for the study. A sub-sample from a larger cohort, recruited as previously described was selected for this study. In brief, 1095 households were recruited for a cluster randomized controlled community trial [22]. Subjects for this study were recruited from the control arm. Children who had a medical/birth defect that affected eating and normal growth were excluded (e.g., cerebral palsy). Data were collected from March to July 2017. The FA variation from a previously reported study in Tanzania was used to run an *a priori* sample size calculation for multiple regression based on an estimated small effect size of 0.05 and significance level *p* = 0.05. This indicated that 220 participants would yield statistical power of 80% [23]. Only 209 children were enrolled, reducing the power to 78%.

### 2.3. Ethical Standards Disclosure

This study was conducted according to the guidelines laid down in the Declaration of Helsinki and all procedures involving human subjects/patients were approved by the Ethics review board at McGill University Canada (IRB #180-1013), the Institutional Review Board at Michigan State University (IRB #16-557) and the Nogouchi Memorial Institute for Medical Research ethics committee (IRB #027/13-14). Written informed consent was obtained from the parent/caregiver of all children. A script of the written consent was read and translated in Twi, Krobo and Ewe to the parents or caregivers of the children. The parent or caregiver of the participating child gave consent prior to the child’s participation. The parents or caregivers’ thumb printed/signed the consent document to give consent. They were assured that participation was voluntary and confidential and that their information would remain anonymous. 

### 2.4. Anthropometric Measurements

Heights of all participants were measured to the nearest 0.1 cm with a ShorrBoard stadiometer (Weigh and Measure LLC, Olney, MD, USA). Weight was measured using a digital bathroom scale to the nearest 0.1 kg (Tanita BMB-800, Tokyo, Japan). The average of two height and weight measurements were recorded. The date of birth and sex was recorded from the child’s health card or birth certificate. Height, weight, date of birth and sex data were entered into World Health Organization (WHO) Anthro [24] (WHO, Geneva, Switzerland) and WHO AnthroPlus [25] software (WHO, Geneva, Switzerland) to calculate height-for-age z-scores (HAZ), weight-for-age z-scores (WAZ), weight-for-height z-scores (WHZ) and BMI-for-age z-scores (BAZ).

### 2.5. Blood Fatty Acid Assessment

Capillary blood sample (40 μL) was obtained by puncturing the middle finger using a sterile single-use lancet, as previously described by Jumbe et al. [23,26]. The first drop of blood was wiped with a sterilized dry pad. The drops of blood were then collected onto the dried blood spot cards, which are pre-treated with anti-oxidant cocktail. The cards were stored in a dry, cool environment and shipped to the USA for FA analysis by OmegaQuant Analytics, LLC (Sioux Falls, SD, USA). On average, the time from sample collection time to arrival to the USA was 8 days. The samples were stored at −80 °C till they were analyzed as previously described [27,28,29]. Briefly, the cards were punched and combined with the derivatizing reagent (boron trifluoride in methanol (14%), toluene and methanol (35:30:35 parts)). The mixture was shaken and heated at 100 °C for 45 min. Forty parts of both hexane and distilled water were added after the mixture had cooled. The mixture was vortexed and then separated into distinct layers. An aliquot of the hexane layer that contained the FA methyl esters was extracted. FA analysis was performed using a standard panel of fatty acids as previously described for consistency [30,31,32]. Unless otherwise stated, whole blood FA proportions are expressed as a percent of total identified FAs.

### 2.6. Hemoglobin and Malaria Status

A hemocue photometer (HemoCue 301, Angelholm, Sweden) was used to determine the hemoglobin concentration. The malaria status was determined using an antigen-based malaria rapid diagnostic test (RDT) kit (Standard Diagnostic Inc., Kyonggi, Korea). These tests were conducted using additional drops of blood from the same punch. 

### 2.7. Dietary Intake Assessment

Using a structured questionnaire, qualitative intake of various foods consumed by subjects within 24 h prior to blood sample collection was assessed. To gauge dietary diversity, the foods that were of interest include fish and seafood, dairy and meats, as these foods could begin to identify dietary sources of long chain PUFAs. 

### 2.8. Data reduction and Statistical Analyses

Means and standard deviations were calculated for descriptive analysis. FA and growth variables were tested for and normally distributed based on the WHO standard population and definitions of moderate and severe stunting, wasting and underweight, percentages were calculated [27]. The FA values were expressed as percent composition of total blood FAs. Total n-3 FA proportions were calculated as ∑ (ALA + EPA + docosapentaenoic acid n-3 + DHA); total n-6 FA proportions were calculated as ∑ (LA + γ-linolenic acid (GLA) + eicosadienoic + dihomo-γ-linolenic (DGLA) + AA + docosatetraenoic acid + docosapentaenoic acidn-6); total n-9 FA proportions were calculated as ∑ (oleic acid + eicosenoic acid + Mead acid + nervonic acid); total saturated FA proportions were calculated as ∑ (myristic acid + palmitic acid + stearic acid + arachidic acid + behenic acid + lignoceric) acid; total MUFA proportions were calculated as ∑ (palmitoleic acid + oleic acid + palmitelaidic acid + nervonic acid + elaidic acid + eicosenoic acid). The T/T ratio was calculated from the ratio of Mead acid and AA [28]. The FA product/precursor ratio was used to estimate the desaturase activity [29] as follows: D5D (delta-5-desaturase) = AA/DGLA; D6D (delta-6-desaturase) = DGLA/LA. The omega-3 index was calculated as previously described based on the sum of EPA and DHA in erythrocyte membranes and then expressed as a percentage of total erythrocyte FA since DBS cards collect whole blood and not only erythrocytes [28]. 

Blood FA composition of children who were stunted and those who were not, as well as FA levels of our previously published northern Ghana children (*n* = 307) [29] versus these data for southern Ghana children, were compared using two sample Student’s *t*-test. Normal probability plots for all FAs were assessed to verify the validity of reporting mean FA levels and the use of regression analysis. Hemoglobin was used as a covariate as it was significantly associated with HAZ and WAZ (*p* ≤ 0.01). Malaria was also adjusted for in the model as a significant percentage (11%) of the children tested positive to RDT/malaria test. Because high collinearity among the FAs caused tolerance levels <0.1 and gave variance inflation factors greater than 10 when all the FAs were entered into a single model, we used single linear regressions to analyze the association between blood FA levels and growth. Regression models consisted of the dependent variable (HAZ, WAZ, WHZ, or BAZ) and were adjusted for each FA, the main exposure variable. Hb and Malaria were included as adjustment variables. (e.g., HAZ = FA + Hb + malaria). Regression models were not adjusted for sex as there were few significant associations between FAs and sex and the regression values were unaffected when evaluated with sex adjustment. 

Next, principal component analysis was used to generate FA patterns, by reducing the number of variables and allowing highly correlated variables to be assessed simultaneously. Exploratory factor analysis was carried out using the psych package [33]. Briefly, scree plot was used to determine top four factors based on eigenvalues [18]. Varimax rotation was used for the orthogonal transformation of the factor loading matrix. This procedure assigns a correlation coefficient for each of the four factors. FAs that correlated with factors *r* ≥ 0.5 were considered strongly correlated with the factor, regardless of sign. Factor loading scores were generated for each child and used to calculate linear regressions using factors as predictors to determine relationships between these factors and growth variables. The regressions were calculated for the dependent variable was used in a model as HAZ, or WAZ = Hb + malaria + Factor. *p*-values were considered significant if *p* ≤ 0.05. Statistical analyses were conducted using R software (R version 3.3.0, Vienna, Austria) and SPSS version 24 (IBM, New York, NY, USA).

## 3. Results

### 3.1. Subject Characteristics

The demographic information for the subjects is presented in Table 1. The mean age of the children was around 3 years and there were slightly more males (52%) than females (48%). The average height of the participants was around 92 cm and the average weight was around 13 kg. The mean hemoglobin concentration was about 11 g/dL. A positive malaria test was detected in 11% of the children. The mean HAZ, WAZ, WHZ and BAZ are −1.4, −1.0, −0.4 and −0.3 respectively. The standard deviations of the HAZ, WAZ, WHZ and BAZ distributions were relatively constant and close to the expected value of 1.00 (range: 0.91 to 1.01). Using the WHO guidelines and criteria [27], 22% of the children were stunted, 13% were underweight, 3% malnourished and 3% were wasted. (Table 2). 

### 3.2. Fatty Acid Levels in Whole Blood

Since FA levels were normally distributed, mean levels for overall, stunted and non-stunted children in this study are shown in Table 3. Approximately 10.6% of all children in the study had whole blood T/T ratio greater than 0.02 and 4.5% had Mead acid levels above 0.21%. For all 209 children enrolled in the study, the average percent of whole blood measured was 40.2% total saturated FAs, 23.3% total monounsaturated FAs, 29.1% total n-6 and 7.2% total n-3 FAs. The main components of n-6 and n-3 FAs were LA and DHA, accounting for 16.7% and 5.1% of whole blood, respectively. Overall, the mean blood FAs percent of total did not differ between stunted and non-stunted children, except for DGLA, that was significantly higher in the stunted children compared to those who were not stunted. Of interest, there was no significant difference between Mead acid when comparing stunted versus non-stunted children. Although not significant, children who were stunted had lower blood levels of DHA (4.95%) than those who were not stunted (5.13%) or the overall group (5.09%). Children who were stunted tended to have lower estimated delta-5 (*p* = 0.054) and had higher estimated delta-6 (*p* = 0.027) desaturase activity ratios. However, no significant difference was found between T/T ratio of both groups.

### 3.3. Relationships or Associations between Fatty Acids and Growth Parameters 

Table 4a shows the results of the regression analysis between HAZ and selected FAs. Regression analysis between WAZ and FAs are also shown in Table 4a. Regression analysis demonstrates what a unit increase in a given growth variable has on a given FA level. There was no significant association between FAs and WAZ and HAZ except for total saturated fats (*p* = 0.08) which was approaching a significant inverse association with HAZ. A significant regression would show that a unit increase in HAZ score (towards stunting) is associated with a 7% decrease total saturated FA level. Table 4b shows a regression between WHZ, as well as BAZ and some selected FAs. With the exception of LA which was inversely related with WHZ approaching significance (*p* = 0.08) and BAZ (*p* = 0.07), all other FAs were not significantly associated with any of the growth parameters. It is notable that a full model including all 25 single FAs analyzed as well as Hb concentrations and malaria status was not significant for HAZ (*r*^2^ = 0.091, *p* = 0.856) or other growth parameters.

### 3.4. Factor Analysis

Due to the FAs being highly correlated, factor analysis was utilized to identify FA groups related to stunting (HAZ) or underweight (WAZ). Factor loadings (Table 5) show the correlation between individual FAs and the respective factor. Factor 1 is mainly driven by n-3 fatty acids EPA, DPA and DHA. Factor 2 was mainly driven by saturated FAs, while factors 3 and 4 were driven by n-6 FAs. Multiple linear regression between these four factors and HAZ, as well as WAZ are shown in Table 6. The overall models were not significant for either HAZ (*p* = 0.798) or WAZ (*p* = 0.953). None of the four factors was significantly associated with HAZ or WAZ in either models.

### 3.5. Mean Fatty Acid Levels for Northern and Southern Ghana, Compared

Next, we compared the mean FA levels between our previously published data from northern Ghanaian children [34] to these data from southern Ghanaian children. Interestingly, there were highly significant differences in mean whole blood percent for most of the FAs (*p* < 0.001; Table 7). Specifically, the mean whole blood levels of n-3 FAs (such as ALA, EPA, DHA, DPA n-3 and omega-3 index) were significantly higher (*p* < 0.001) in the southern Ghana population than in the northern Ghana population. Mean level of DHA and the omega-3 index, for example, were 2.62% and 4.55%, respectively in the northern Ghana population compared to 5.09% and 8.03% in the southern Ghana population. Further, the mean whole blood n-6 FAs LA, AA, DGLA, Eicosadienoic acid, DTA and DPA n-6 and were higher in the northern Ghana population (*p* < 0.001). The T/T ratio and Mead acid were higher in northern Ghana population than in the southern Ghana population (*p* < 0.001; Table 7). This indicates a higher rate of EFA deficiency in the north compared to the south.

### 3.6. Dietary Intake of Protein and Fat-Rich Foods 

Records of protein intake from the dietary intake assessment in this southern Ghana study show that 93% of the children consumed fish or seafood 24 h prior to data collection. The records also show that 24%, 18% and 17% of the children consumed poultry, eggs and dairy products, respectively.

## 4. Discussion

The objective of this study was to describe the whole blood FA levels of southern Ghanaian children and to determine the association between FAs and growth parameters. The prevalence of stunting in our sample was 22% which is higher than the 17% stunting rate reported by the GDHS in the Eastern region of Ghana. However, the stunting rate in this population is lower than the rate reported for northern Ghana (29%) [35]. The prevalence of EFA deficiency was 4.6% and 10.6% based on the Mead acid levels and T/T ratio, respectively. These values are both lower than what was previously reported in Tanzania [23] and thus indicated a lower EFAD in southern Ghana. With the exception of total saturated FAs and LA which showed a trending significance with some growth parameters, none of the FAs were associated with growth parameters. When the blood levels of FAs from this study were compared to previously reported data from northern Ghana, most n-3 FAs levels were significantly higher in the southern Ghana population (*p* < 0.001) and most n-6 FAs level were significantly higher in the northern Ghana population (*p* < 0.001). 

Mean omega-3 index in the southern Ghana population is significantly higher (*p* < 0.001) than what was reported in an earlier study in northern Ghana [34]. Whole blood levels of DHA and EPA are highly dependent on dietary intake. Although, there is conversion of ALA to its metabolites including EPA and DHA, there is limited conversion of ALA to DHA in mammalian species [36]. It is therefore likely that the high EPA and DHA levels were obtained from the diet and contribute to the omega-3 index. While the intake of carbohydrates are similar across populations in Ghana [37], it is likely that location or local culture may contribute to limited access of foods rich in DHA and EPA. Over 90% of the subjects consumed fish and seafood in the 24 h prior to data collection. This consumption pattern is higher when compared to data collected by the GDHS which states that only children between the ages of 6–23 months, only 47.6% of them consumed meat, fish and poultry 24 h prior to data collection [1]. As stated earlier, apart from agriculture and forestry, fishing is one economic activity that is common among inhabitants of Upper Manya Krobo district [21]. As a primary economic activity in the communities, fishing can increase accessibility and hence consumption of fish compared to the northern region where the inhabitants are predominantly farmers and traders living in mostly landlocked communities [38]. Geographic difference between northern and southern Ghana may contribute to the kinds of food being grown, harvested and hence variation in dietary composition of the foods being consumed [37]. These geographic differences in fish intake, for example, could explain the regional variations of omega-3 index across the two populations. The omega-3 index for southern Ghanaian children was also higher than values reported for Australian school-aged children [39], United Kingdom children aged 7–9 years [40], Tanzanian children 2–6 years [41] and in European children 3–8 years [42]. The reasons for these variations is likely attributable to the dietary diversity in the various populations. 

Omega-6 FAs have crucial roles in growth. A previous study from our group reported an association of n-6 PUFAs and linear growth in Tanzanian children [23]. The LC n-6 PUFAs such as AA modulate varied physiological responses such as cell growth and differentiation in conditions that involve altered cellular proliferation [6]. Compared to the Northern Ghana population, stunting was lower in this population and so were the n-6 FA levels. Northern Ghana population has higher stunting and reported a higher level of n-6 FAs. The significant difference in lower delta-5 and higher delta-6 estimated activities in the stunted compared to non-stunted groups can be attributed to significant difference in DGLA levels among both groups. Lower levels of AA in the stunted group, which along with higher DGLA would account for lower activity of delta-5 (AA/DGLA) but not of delta-6 (DGLA/LA) desaturase. A decrease in dietary consumption of n-6 precursor LA in this population may lead to a decrease in n-6 FA metabolites. Lower dietary consumption of n-6 FAs could further lead to the suppression of the n-6 FA pathways by the high amounts of dietary n-3 FAs. These findings support previous observations that the n-3 and n-6 FA pathways are competitive and an increase in dietary intake of one could suppress the other [43]. 

The exact mechanism of n-3 FAs in growth remains unclear; however, their roles in growth is undisputed [44]. Omega-3 FAs have beneficial roles in bone metabolism, by increasing bone formation that affects peak bone mass and reducing bone loss [45]. This is because n-3 FAs reduce inflammatory cytokines, increase calcium resorption and enhance calcium levels [45], all roles that contribute to growth in children. The southern Ghanaian children who participated in this study were reported to have fish or seafood consumption. Fish and seafood are important sources of EPA and DHA. They also contain high quality proteins, amino acids, vitamins and minerals [46,47]. In addition, fish and seafood have high amounts of essential amino acids such as leucine, lysine and tryptophan [48]. The lower levels of stunting observed in this region of Ghana could possibly be due to quality dietary protein intake of fish and seafood in this population [21]. In support, a recent study by Semba et al. reported that child stunting was associated with circulating amino acids and also reported that stunted children had a limited amount of tryptophan and lysine in their diet [49]. The intake of high quality proteins in this population may reduce the prevalence of protein energy malnutrition leading to enhanced linear growth and reduced stunting in this population.

This is the first study to assess whole blood FAs in southern Ghanaian children 2–6 years old. This study utilized whole blood FA biomarkers and analyzed the association with growth. The use of a validated dried blood spot collection and blood transport system made the study logistically easier to conduct. This method was also successfully used in a similar study in northern Ghana [34] and in Tanzania [23]. Aside from these strengths, this study also has limitations that should be noted. We did not collect information on extensive dietary intake hence we were unable to account for the role of other nutrients on growth parameters. Iodine, zinc and other vitamins and minerals have roles to play in growth but the study could not account for all these nutrients. In the population, children who were seen as underweight may have had adjustments by their community health center to include animal source diets to meals. We did not collect information on children who may have had dietary adjustments. The collection of blood samples took place throughout the day and the subjects were not required to fast. Since the FA intake across each population was similar, this was not expected to have an effect on the variability of the whole blood FA composition. In addition, the majority of fatty acids in a dried blood spot (DBS) sample are in cell membranes and thus are not affected by recent meals or a short period of time. Finally, this study cannot be generalized to the whole Ghanaian population because the study was performed in one area in southern Ghana and dietary intake of foods may differ across locations in Ghana. 

## 5. Conclusions

This study assessed whole blood levels of EFAs and their relationship with growth parameters in southern Ghanaian children 2–6 years of age. Deficiency of EFA was observed in 10.64% of children while stunting was observed in 22% of all children; no association was noted between whole blood FA and growth parameters. These data suggest that factors other than EFA deficiency may influence stunting in this population. There is, therefore, the need for further studies to explore other nutrients and other factors that could be related to stunting in this population. Further, over 90% of all subjects reported dietary intake of fish and seafood prior to blood collection and this could decrease the risk of malnutrition and EFA deficiency as a potential cause of stunting in this population. Interestingly, the whole blood n-3 FAs were much higher in this population than that reported in children in other geographical areas. The intake of fish in this population could account for low stunting levels in this population consistent with previous studies demonstrating that increasing consumption of fish and fish oil intake can increase linear growth in children [47,49]. 

## Figures and Tables

**Table 1:** Demographic characteristics of the 209 children enrolled.

Characteristic	Mean ± SD		
	Overall (*n* = 209)	Male (*n* = 108)	Female (*n* = 101)
Age (months)	38.3 ± 9.9	38.5 ± 9.9	38.2 ± 10.1
Height (cm)	91.5 ± 7.1	91.9 ± 7.0	90.9 ± 7.2
Weight (kg)	12.8 ± 2.1	13.1 ± 1.9	12.5 ± 2.2
HAZ	−1.4 ± 0.9	−1.4 ± 0.9	−1.3 ± 0.9
BAZ	−0.3 ± 1.0	−0.2 ± 0.9	−0.4 ± 1.1
WAZ	−1.0 ± 0.9	−1.0 ± 0.9	−1.1 ± 1.1
WHZ	−0.4 ± 1.0	−0.3 ± 0.9	−0.5 ± 1.1
Hb (g/dL)	10.9 ± 1.4	10.8 ± 1.5	11.0 ± 1.3

HAZ, height-for-age z-score; BAZ, BMI-for-age z-score; WAZ, weight-for-age z-score; WHZ, weight-for-height z-score; Hb, hemoglobin.

**Table 2:** Percentage of children in each category of growth parameters as defined by WHO (*n* = 209) *.

Growth Variable	Based on	Severe	Moderate	Unaffected
		(<−3SD)	(−3 ≤ SD ≤ −2)	t > −2SD
Stunting	HAZ	4.3%	17.7%	78.0%
Malnutrition	BAZ	1.0%	1.9%	97.1%
Underweight	WAZ	2.4%	10.5%	87.1%
Wasting	WHZ	1.0%	2.4%	96.6%

HAZ, height-for-age z-score; BAZ, BMI-for-age z-score; WAZ, weight-for-age z-score; WHZ, weight-for-height z-score. * The WHO definitions of moderate and severe stunting, malnutrition, underweight and wasting were applied to the data [33].

**Table 3:** Mean fatty acid levels of overall, stunted and non-stunted children in southern Ghana (Values expressed as mean ± standard deviation).

Fatty Acid (% Total)	Overall (*n* = 209)	Stunted (*n* = 46)	Not Stunted (*n* = 163)	*p*-Value
C14:0 Myristic Acid	0.82 ± 0.59	0.76 ± 0.41	0.84 ± 0.63	0.460
C16:0 Palmitic Acid	25.7 ± 1.62	25.8 ± 1.53	25.6 ± 1.65	0.592
C18:0 Stearic Acid	11.9 ± 1.16	12.1 ± 1.14	11.9 ± 1.16	0.209
C20:0 Arachidic Acid	9.18 ± 1.56	0.31 ± 0.06	0.31 ± 0.06	0.752
C21:0 Behenic Acid	0.84 ± 0.41	0.59 ± 0.16	0.56 ± 0.15	0.215
C24:0 Lignoceric Acid	0.90 ± 0.34	0.95 ± 0.39	0.88 ± 0.32	0.251
Total sat	40.2 ± 1.54	40.5 ± 1.46	40.1 ± 1.56	0.108
C16:1 Palmitoleic Acid	0.84 ± 0.41	0.88 ± 0.50	0.82 ± 0.39	0.411
C18:1 Oleic Acid	21.0 ± 2.68	20.8 ± 2.56	21.0 ± 2.72	0.596
C20:1 Eicosenoic Acid	0.34 ± 0.14	0.37 ± 0.13	0.33 ± 0.14	0.156
C24:1 Nervonic Acid	0.83 ± 0.30	0.87 ± 0.30	0.82 ± 0.29	0.248
Total MUFA	23.3 ± 2.70	23.2 ± 2.62	23.3 ± 2.73	0.818
C18:3n3 alpha-Linolenic Acid (ALA)	0.25 ± 0.10	0.27 ± 0.12	0.25 ± 0.09	0.645
C20:5n3 Eicosapentaenoic Acid (EPA)	0.80 ± 0.35	0.74 ± 0.23	0.81 ± 0.38	0.220
C20:5n3 Docosapentaenoic Acid (DPA n-3)	1.01 ± 0.21	1.04 ± 0.21	1.00 ± 0.21	0.364
C22:6n3 Docosahexaenoic Acid (DHA)	5.09 ± 0.98	4.95 ± 0.95	5.13 ± 0.98	0.263
Total n-3	7.15 ± 1.34	7.00 ± 1.19	7.19 ± 1.37	0.379
Omega-3 Index	8.03 ± 1.37	7.80 ± 1.23	8.09 ± 1.40	0.204
C18:2n6 Linoleic Acid (LA)	16.7 ± 1.92	16.6 ± 2.43	16.7 ± 1.76	0.589
C18:3n6 γ-Linolenic Acid (GLA)	0.21 ± 0.09	0.20 ± 0.10	0.21 ± 0.08	0.610
C20:2n6 Eicosadienoic Acid	0.03 ± 0.05	0.25 ± 0.06	0.25 ± 0.05	0.156
C20:3n6 Dihomo-γ-linolenic Acid (DGLA)	1.24 ± 0.23	1.30 ± 0.25	1.22 ± 0.22	0.048
C20:4n6 Arachidonic Acid (AA)	9.18 ± 1.56	9.09 ± 1.29	9.21 ± 1.64	0.645
C22:4n6 Docosatetraenoic Acid (DTA)	1.03 ± 0.25	1.09 ± 0.22	1.02 ± 0.26	0.118
C22:5n6 Docosapentaenoic Acid (DPA n-6)	0.45 ± 0.13	1.02 ± 0.26	0.44 ± 0.13	0.262
Total n-6	29.1 ± 2.60	29.0 ± 2.71	29.1 ± 2.57	0.779
C20:3n9 Mead acid	0.09 ± 0.01	0.09 ± 0.07	0.09 ± 0.06	0.920
Total n-6/Total n-3 ratio	4.19 ± 0.81	4.26 ± 0.84	4.18 ± 0.08	0.561
Delta-6-Desaturase (D6D)	0.08 ± 0.02	0.08 ± 0.02	0.07 ± 0.02	0.027
Delta-5-Desaturase (D5D)	7.61 ± 1.68	7.22 ± 1.48	7.72 ± 1.72	0.054
T/T ratio	0.01 ± 0.01	0.01 ± 0.01	0.01 ± 0.01	0.960

Blood samples of southern Ghanaian children were collected on Dried Blood spot cards and fatty acid levels were analyzed by OmegaQuant LLC. Mean FA levels are reported as data was normally distributed. Students *t*-test was used to compare individual fatty acid levels between stunted and non-stunted groups to identify significant differences in FA levels. *p*-value ≤0.05 shows a significant difference.

**Table 4:** (**a**) Regression results between HAZ, WAZ and selected fatty acids. (**b**) Regression results between WHZ, BAZ and selected fatty acids.

(a)				
Fatty Acid	HAZ		WAZ	
	B ± SE	*p*-Value	B ± SE	*p*-Value
C14:0 Myristic Acid	−0.09 ± 0.11	0.378	−0.10 ± 0.12	0.382
C16:0 Palmitic Acid	−0.04 ± 0.04	0.384	0.00 ± 0.04	0.829
C18:0 Stearic Acid	−0.04 ± 0.06	0.504	−0.01 ± 0.06	0.921
Total sat	−0.07 ± 0.04	0.084	−0.03 ± 0.05	0.564
C16:1 Palmitoleic Acid	−0.08 ± 0.15	0.610	0.01 ± 0.16	0.957
C18:1 Oleic Acid	−0.00 ± 0.02	0.924	0.01 ± 0.03	0.808
C20:1 Eicosenoic Acid	−0.78 ± 0.46	0.089	−0.48 ± 0.49	0.324
C24:1 Nervonic Acid	0.04 ± 0.22	0.860	0.14 ± 0.23	0.535
Total MUFA	−0.01 ± 0.02	0.795	−0.01 ± 0.03	0.781
C18:3n3 alpha-Linolenic Acid (ALA)	−0.57 ± 0.66	0.386	0.32 ± 0.70	0.651
C20:5n3 Eicosapentaenoic Acid (EPA)	0.00 ± 0.18	0.999	0.04 ± 0.19	0.846
C20:5n3 Docosapentaenoic Acid (DPA n-3)	−0.20 ± 0.31	0.505	0.20 ± 0.33	0.529
C22:6n3 Docosahexaenoic Acid (DHA)	0.06 ± 0.07	0.390	0.03 ± 0.07	0.717
Total n-3	0.02 ± 0.05	0.646	0.02 ± 0.05	0.653
C18:2n6 Linoleic Acid (LA)	0.04 ± 0.03	0.220	−0.02 ± 0.04	0.679
C18:3n6 γ-Linoleic Acid (GLA)	0.77 ± 0.68	0.254	0.82 ± 0.72	0.255
C20:2n6 Eicosadienoic Acid	0.50 ± 1.25	0.688	0.75 ± 1.33	0.575
C20:3n6 Dihomo-γ-linolenic Acid (DGLA)	0.05 ± 0.28	0.868	0.31 ± 0.30	0.305
C20:4n6 Arachidonic Acid (AA)	0.01 ± 0.04	0.747	−0.01 ± 0.05	0.899
C22:4n6 Docosatetraenoic Acid (DTA)	−0.27 ± 0.25	0.291	0.02 ± 0.27	0.948
C22:5n6 Docosapentaenoic Acid (DPA n-6)	−0.13 ± 0.48	0.780	0.40 ± 0.51	0.434
Total n-6	0.03 ± 0.03	0.319	−0.01 ± 0.03	0.832
Total n-6/Total n-3 ratio	0.02 ± 0.08	0.772	−0.07 ± 0.08	0.423
Omega-3 Index	0.03 ± 0.05	0.484	0.02 ± 0.05	0.724
T/T ratio	5.51 ± 9.21	0.551	−4.53 ± 9.83	0.645
Single linear regressions were used to analyze association of blood FA levels and growth. Because many FA are highly correlated, single regression analysis was necessary. Each growth variable (left; HAZ, right; WAZ) was regressed with individual fatty acids, adjusting for hemogloblin and malaria. Model: HAZ = fatty acid + hemoglobin + malaria; WAZ = fatty acid + hemoglobin + malaria.				
(**b**)				
**Fatty Acid**	**WHZ**		**BAZ**	
	**B ± SE**	***p*** **-Value**	**B ± SE**	***p*** **-Value**
C14:0 Myristic Acid	−0.10 ± 0.12	0.412	−0.07 ± 0.12	0.547
C16:0 Palmitic Acid	0.02 ± 0.05	0.652	0.03 ± 0.05	0.550
C18:0 Stearic Acid	0.03 ± 0.06	0.587	0.03 ± 0.06	0.633
Total sat	0.03 ± 0.05	0.53	0.03 ± 0.05	0.407
C16:1 Palmitoleic Acid	0.12 ± 0.17	0.499	0.10 ± 0.17	0.557
C18:1 Oleic Acid	0.02 ± 0.03	0.559	0.02 ± 0.03	0.569
C20:1 Eicosenoic Acid	0.02 ± 0.51	0.974	0.11 ± 0.51	0.831
C24:1 Nervonic Acid	0.19 ± 0.24	0.428	0.19 ± 0.24	0.441
Total MUFA	0.01 ± 0.03	0.425	0.02 ± 0.03	0.436
C18:3n3 alpha-Linolenic Acid (ALA)	0.88 ± 0.73	0.231	1.06 ± 0.73	0.151
C20:5n3 Eicosapentaenoic Acid (EPA)	0.02 ± 0.20	0.903	0.03 ± 0.20	0.866
C20:5n3 Docosapentaenoic Acid (DPA n-3)	0.46 ± 0.34	0.181	0.49 ± 0.34	0.149
C22:6n3 Docosahexaenoic Acid (DHA)	−0.02 ± 0.07	0.796	−0.03 ± 0.07	0.716
Total n-3	0.01 ± 0.05	0.886	0.01 ± 0.05	0.911
C18:2n6 Linoleic Acid (LA)	−0.06 ± 0.04	0.079	−0.07 ± 0.04	0.073
C18:3n6 γ -Linoleic Acid (GLA)	0.78 ± 0.75	0.304	0.54 ± 0.75	0.477
C20:2n6 Eicosadienoic Acid	0.50 ± 1.39	0.674	0.51 ± 0.39	0.716
C20:3n6 Dihomo- γ -linolenic Acid (DGLA)	0.52 ± 0.39	0.095	0.52 ± 0.31	0.098
C20:4n6 Arachidonic Acid (AA)	−0.03 ± 0.05	0.564	−0.03 ± 0.05	0.533
C22:4n6 Docosatetraenoic Acid (DTA)	0.27 ± 0.28	0.333	0.28 ± 0.28	0.277
C22:5n6 Docosapentaenoic Acid (DPA n-6)	0.66 ± 0.53	0.217	0.72 ± 0.53	0.175
Total n-6	−0.04 ± 0.03	0.186	−0.04 ± 0.03	0.173
Total n-6/Total n-3 ratio	−0.12 ± 0.09	0.180	−0.12 ± 0.09	0.178
Omega-3 Index	−0.01 ± 0.05	0.862	−0.01 ± 0.05	0.806
T/T ratio	−8.27 ± 10.2	0.421	11.4 ± 10.2	0.267
Single linear regressions were used to analyze association of blood FA levels and growth variables due to high collinearity among FAs. Each growth variable (left; WHZ, right; BAZ) was regressed with individual fatty acids, adjusting for hemogloblin and malaria. Model: WHZ = fatty acid + hemoglobin + malaria; BAZ = fatty acid + hemoglobin + malaria).				

**Table 5:** Factor analysis of fatty acids.

Fatty Acid	Factor 1	Factor 2	Factor 3	Factor 4
DHA	**0.78**	0.27	0.15	−0.01
EPA	**0.76**	−0.06	−0.20	−0.11
DPA	**0.70**	0.17	0.30	0.02
Oleic	**−0.70**	−0.37	−0.37	0.19
AA	**0.63**	0.12	**0.54**	−0.05
GLA	−0.36	0.04	0.33	0.36
Behenic	0.04	**0.91**	0.00	−0.01
Lignoceric	0.09	**0.87**	0.12	−0.18
Nervonic	0.05	**0.82**	0.14	−0.18
Arachidic	0.15	**0.63**	−0.24	0.26
Stearic	0.44	**0.55**	0.38	0.30
ALA	−0.06	−0.18	−0.18	−0.05
Docosatetraenoic	0.12	0.17	**0.77**	0.10
DGLA	−0.05	0.06	**0.76**	0.11
DPA n-6	0.11	−0.16	**0.74**	−0.07
Eicosadienoic	0.05	−0.05	**0.44**	0.09
Myristic	0.11	−0.17	−0.38	0.04
LA	−0.02	−0.06	−0.02	**–0.75**
Mead	−0.38	0.05	0.34	**0.57**
Eicosenoic	0.06	0.42	−0.26	**0.56**

Varimax rotated factor-loading matrix generated using the R-package psych. Factors named based on majority of highly correlated FAs. Numbers displayed represent each FA correlation with its respective factor. Correlations ≥ 0.50 are bolded.

**Table 6:** HAZ and WAZ regressed on calculated factors (HAZ or WAZ = Factor + Hb + malaria).

Factor	HAZ		WAZ	
	Beta	*p*-Value	Beta	*p*-Value
Hb	−0.02	0.617	−0.01	0.887
Malaria	−0.001	0.316	−0.001	0.378
Factor 1	−0.04	0.591	−0.02	0.826
Factor 2	0.003	0.968	0.01	0.929
Factor 3	0.03	0.602	0.06	0.406
Factor 4	−0.09	0.180	−0.03	0.628

HAZ, height for age z-score; WAZ, weight for age z-score; Hb, hemoglobin.

**Table 7:** Mean percent FA levels for northern and southern Ghanaian children.

Fatty Acid (% of Total)	Southern Ghana *n* = 209	Northern Ghana *n* = 307	*p*-Value
C18:3n3 alpha-Linolenic Acid (ALA)	0.25 ± 0.10	0.18 ± 0.12	<0.001
C20:5n3 Eicosapentaenoic Acid (EPA)	0.80 ± 0.35	0.22 ± 0.26	<0.001
C20:5n3 Docosapentaenoic Acid (DPA n-3)	1.01 ± 0.21	0.58 ± 0.18	<0.001
C22:6n3 Docosahexaenoic Acid (DHA)	5.09 ± 0.98	2.62 ± 0.64	<0.001
Total n-3	7.15 ± 1.34	3.61 ± 0.91	<0.001
Omega-3 Index	8.03 ± 1.37	4.55 ± 0.92	<0.001
C18:2n6 Linoleic Acid (LA)	16.7 ± 1.92	20.6 ± 1.85	<0.001
C18:3n6 γ -Linoleic Acid (GLA)	0.21 ± 0.09	0.17 ± 0.07	<0.001
C20:2n6 Eicosadienoic Acid	0.25 ± 0.05	0.29 ± 0.07	<0.001
C20:3n6 Dihomo-γ-linolenic Acid (DGLA)	1.24 ± 0.23	1.36 ± 0.26	<0.001
C20:4n6 Arachidonic Acid (AA)	9.18 ± 1.56	10.8 ± 1.61	<0.001
C22:4n6 Docosatetraenoic Acid (DTA)	1.03 ± 0.25	1.69 ± 0.37	<0.001
C22:5n6 Docosapentaenoic Acid (DPA n-6)	0.45 ± 0.13	0.59 ± 0.17	<0.001
Total n-6	29.1 ± 2.60	35.7 ± 2.53	<0.001
n6/n3 ratio	4.20 ± 0.81	10.3 ± 2.00	<0.001
T/T Ratio	0.010 ± 0.007	0.013 ± 0.005	<0.001
Mead acid	0.09 ± 0.06	0.14 ± 0.05	<0.001

FA data from northern Ghana fatty [31] were compared to the Southern Ghana data in this study. Whole blood FA were analyzed similarly and were normally distributed. Thus, a student’s *t*-test was used to compare mean differences in individual FA levels between the two regions.
